# Crimean-Congo hemorrhagic fever virus in tortoises and *Hyalomma aegyptium* ticks in East Thrace, Turkey: potential of a cryptic transmission cycle

**DOI:** 10.1186/s13071-020-04074-6

**Published:** 2020-04-19

**Authors:** Sirri Kar, Sergio E. Rodriguez, Gurkan Akyildiz, Maria N. B. Cajimat, Rifat Bircan, Megan C. Mears, Dennis A. Bente, Aysen G. Keles

**Affiliations:** 1 Galveston National Laboratory, Department of Microbiology and Immunology, Institute for Human Infections and Immunity, University of Texas Medical Branch, Galveston, TX USA; 2 Department of Biology, Tekirdag Namik Kemal University, Tekirdag, Turkey; 3 Department of Pathology, University of Texas Medical Branch, Galveston, TX USA; 4 Faculty of Health Sciences, Marmara University, Istanbul, Turkey

**Keywords:** Crimean-Congo hemorrhagic fever virus, *Hyalomma aegyptium*, Tortoise, Turkey, Thrace, Cryptic transmission cycle

## Abstract

**Background:**

Recent reports have demonstrated the presence of Crimean-Congo hemorrhagic fever virus (CCHFV) genomic material in *Hyalomma aegyptium* ticks feeding primarily on tortoises belonging to the genus *Testudo*. This raises the question if these ticks and their hosts play a role in the natural transmission dynamics of CCHFV. However, the studies are limited, and assessing the relevance of *H. aegyptium* in perpetuating the virus in nature, and a potential spillover to humans remains unknown. This study aimed to detect CCHFV in *H. aegyptium* ticks and their tortoise hosts in the East Thrace region of Turkey, where *H. aegyptium* is the most common human-biting tick and where a high density of tortoises of the genus *Testudo* can be found.

**Methods:**

During the study period, 21 blood samples from different tortoises (2 *T. hermanni* and 19 *T. graeca*), 106 tick pools (containing 448 males, 152 females, 93 nymphs and 60 larvae) collected from 65 tortoises (5 *T. hermanni* and 60 *T. graeca*), 38 adult unfed questing ticks (25 males and 13 females, screened individually) and 14 pools (containing 8 nymphs and 266 larvae) of immature unfed questing ticks collected from the ground were screened for CCHFV genome by nested PCR and partial genomes sequenced.

**Results:**

As a result of the screening of these 179 samples, 17 (9.5%) were detected as positive as follows: 2 of 21 blood samples (9.52%), 13 (containing 18 nymphs in 3 pools, and 52 males and 8 females in 10 pools) of 106 tick pools from tortoises (12.26%), and 2 of 38 adult questing ticks (5.26%). No positive result was determined in 14 pools of immature questing ticks.

**Conclusions:**

Previous studies have shown that reptiles can participate in the transmission of arthropod-borne viruses, but they may contribute to different aspects of the disease ecology and evolution of tick-borne viral pathogens. Our results indicate the presence of CCHFV in questing and feeding *H. aegyptium* ticks as well as tortoise hosts. This may indicate that CCHFV circulates in a cryptic transmission cycle in addition to the primary transmission cycle that could play a role in the natural dynamic of the virus and the transmission to humans.
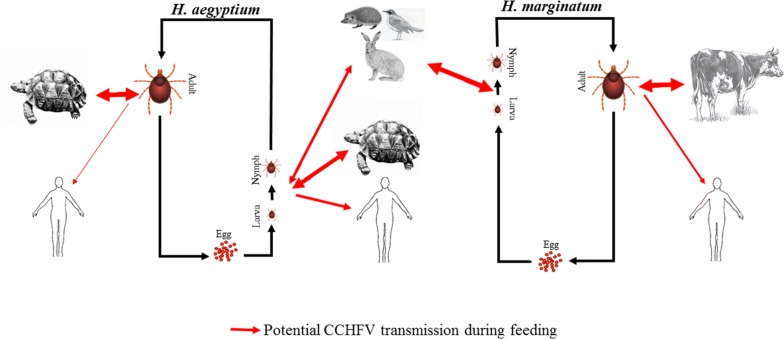

## Introduction

**Fig. 1:**
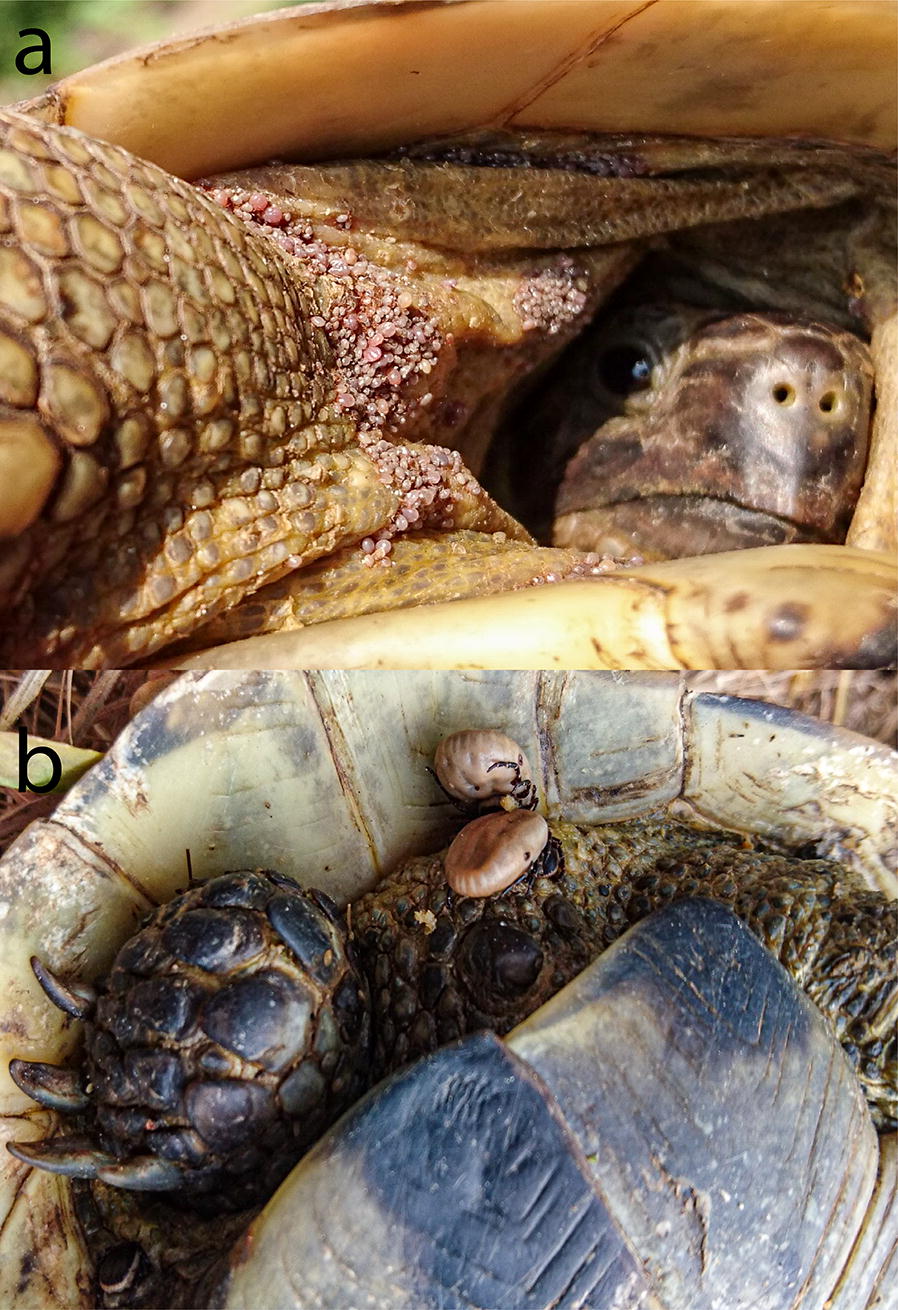
Feeding site preference of *Hyalomma aegyptium* infestation on *T. graeca*. **a***H. aegyptium* immature stages co-feeding on the rostral part of the host. **b***H. aegyptium* adult life stages prefer feeding between the leg and tail on the caudal region of the host

Crimean-Congo hemorrhagic fever (CCHF) is a viral (*Bunyavirales*) zoonoses that is endemic to Africa, the Balkans, the Middle East, and Western Asia, and occurs within countries south of the 50° parallel north, where 3 billion people are globally at risk and approximately 10,000 to 15,000 human cases are estimated to occur with approximately 500 deaths annually [1]. CCHF epidemiology in the Republic of Turkey represents a unique situation, as the first human cases were reported in 2002, and since then case numbers have increased significantly [2]. According to data from Turkey’s Ministry of Health [3], 11,041 human cases with a fatality rate of 4.8% have been recorded from 2002 to the end of 2018. The disease is recorded in almost all parts of the country with different incidences, and most of the cases (*c.*95%) have been reported from the northern plateau of central and eastern Anatolia, especially from Kelkit Valley and its associated extensions (Fig. 1). In Turkey, Crimean-Congo hemorrhagic fever virus (CCHFV) is transmitted to humans predominantly *via* infected tick bites and in some cases, through nosocomial transmission [4].

CCHFV is maintained in nature in a silent transmission cycle between ticks of the genus *Hyalomma* and their hosts, where ticks are considered both the vector and the natural reservoir of the agent. Of those, *H. marginatum* was reported to have the most prominent role (e.g. primary CCHFV transmission cycle) in the Western Palearctic region, including in Turkey in the natural history of the disease [4, 5]. This tick species is a two-host tick in which the immature stages feed on some small or medium-sized animals such as hare, hedgehog, and ground-dwelling birds whereas the adults prefer to feed on ruminants, especially cattle [6].

*Hyalomma aegyptium* (Linnaeus, 1758) is a three-host tick and has an extremely long feeding period compared to most of the other tick species [7]. All stages, but especially adults, are highly host- specific and feed primarily on tortoises. Occasionally *H. aegyptium* immatures are found on other animals such as hedgehogs, other mammals [6, 8, 9] and humans [10–12]. As the result of dependence on tortoise hosts, *H. aegyptium* is found particularly in the Mediterranean region, the Black Sea, and the Middle East, and penetrates eastwards as far as Central Asia, Afghanistan and Pakistan, where tortoise species belong to the genus *Testudo* are found [9, 13]. *Testudo graeca* is the most frequently reported host of *H. aegyptium* [9], but this tick can be encountered on *T. horsfieldii* [14], *T. marginata* (Schoepff, 1789), *T. hermanni* [9], and rarely on *T. kleinmanni* (Lortet, 1883) [15]. As for tick species infesting tortoises, it is known that *H. aegyptium* is the primary tick species infesting Palaearctic tortoises, *Testudo* spp. [9, 16, 17].

Our previous studies have shown that the principle tick species associated with the biting of humans in the Thrace region of Turkey are immature stages of *H. aegyptium* [10, 11]. The frequency of record of this tick species on humans varies from region to region in Turkey, possibly depending on the varying population density of the tick in different regions [10, 18, 19]. It is known that *T. graeca* can be found in all parts of Turkey except for the east part of the Black Sea coastline, while *T. hermanni* is found primarily in the north-west half of the Thrace region [20, 21]. There are no detailed data about distribution and density of the tortoise population in Turkey; however, the reported data related to human-biting *H. aegyptium* seems to be useful for related estimation at this point. For example, in Thrace, Istanbul, other parts of the Marmara Sea basin, and Aegean regions, percentages of larval and nymphal stages of *Hyalomma* in human-biting ticks were reported to vary between 0.7–1.5% and 23.6–68.5% respectively [10, 11, 12, 22]; these stages were morphologically described as *H. aegyptium* in some of the studies [10, 11]. However, these values were 0.2% and 27.1% in Ankara [19], and 0.0–0.5% and 7.9-–21.6% in Kelkit Valley, CCHF hot spot of Turkey [23].

Recently, reports have demonstrated the presence of CCHFV genomic material in *H. aegyptium* as well as CCHFV seroconversion within the ticksʼ primary host, tortoises belonging to the genus *Testudo* [24–27]. Nevertheless, data supporting the relevance of *H. aegyptium* ticks in the role of CCHFV transmission to humans are lacking, and it is unclear if this tick species has any role in the natural dynamics of the virus. This study examined the presence of CCHFV in *H. aegyptium* ticks and their tortoise hosts in the Thrace region of Turkey and sought to understand whether *H. aegyptium* and its tortoise host play a role in the ecology of the CCHFV maintenance in this region. In this study, different life stages of *H. aegyptium* were collected, i.e. questing ticks in the field and ticks feeding on various tortoise hosts (*T. graeca* and *T. hermanni*). Blood was also drawn concurrently from tortoises harboring *H. aegyptium* within this region and examined for CCHFV. Our results indicate the presence of CCHFV in questing and feeding *H. aegyptium* ticks as well as in the tortoise host, potentially indicating a CCHFV cryptic transmission cycle that could play a role in maintaining the virus in nature.

## Methods

### Study area

**Fig. 2:**
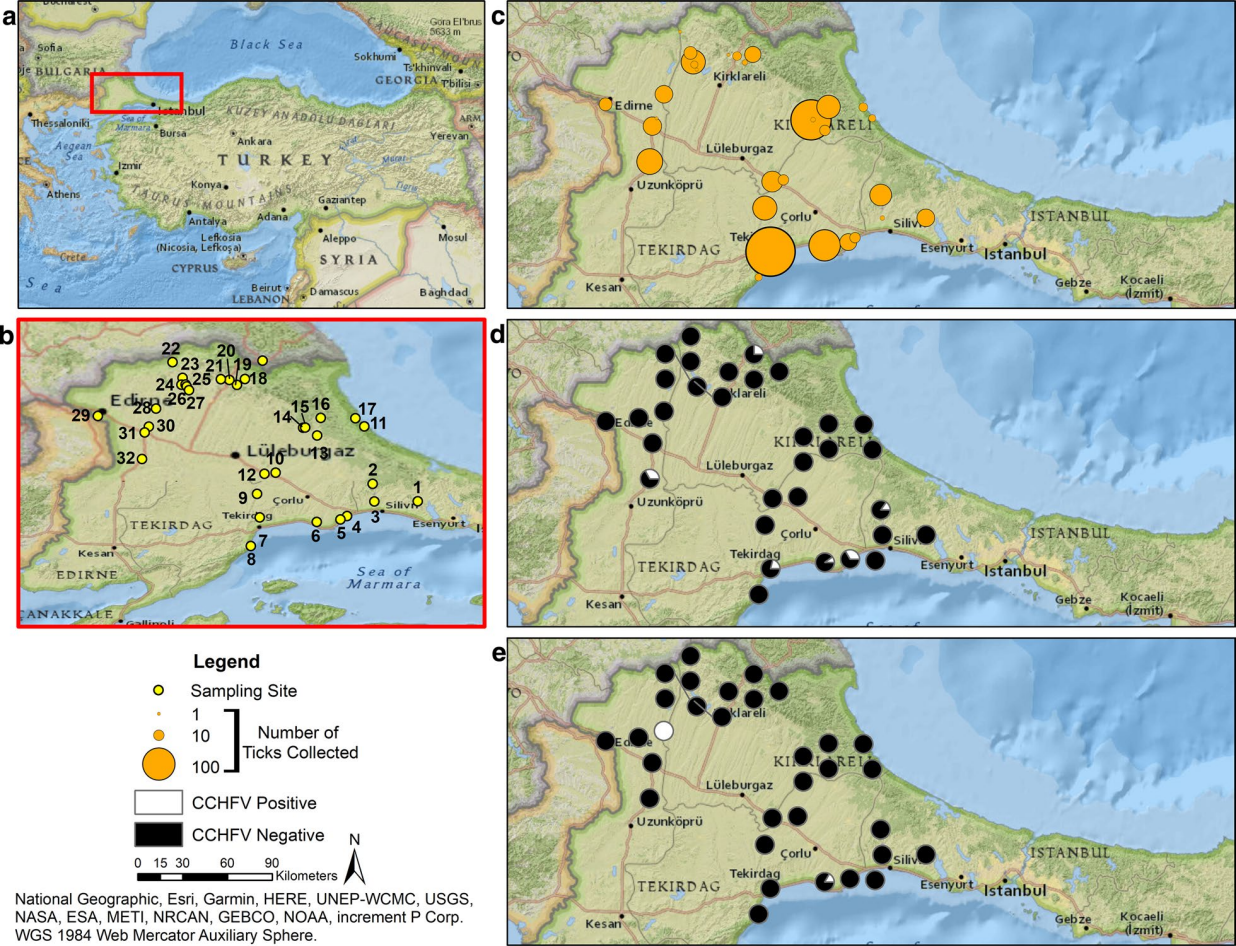
View of the study sites and visual representations of selected data at each sampling site. **a** An overview map of the field study site in the Turkish region of East Thrace. **b** Inset map visualizing 32 sample collection sites in East Thrace. **c** Proportional symbol map of the total number of ticks collected from the field and from tortoises at each sample site. **d** Relative proportion map of tick pools positive (white) and negative (black) for CCHFV by nested RT-PCR at each sample site. **e** Relative proportion map of tortoise blood samples positive (white) and negative (black) for CCHFV by nested RT-PCR at each sample site. Maps were created using ArcGIS® software by Esri. Raw data are provided in Additional file 1: Tables S1 and S2

This study was conducted in the European part of Turkey, known as East Thrace (41° 58′ N, 27° 22′ E at center), which is bordered by Greece, Bulgaria, the Bosphorus Strait, and the Black, Marmara, and Aegean Seas. The western districts of Istanbul Province, Catalca and Silivri, and Tekirdag, Kirklareli and Edirne provinces, were included in this study (Fig 2a, b). According to the records of the Turkish State Meteorological Service [28], there are two distinct geographical conditions within East Thrace: the interior and southern regions contain plains, undulating landscape, and cultivable areas with short and dry vegetation, whereas the north is hallmarked by high mountains and dense, rainy, deciduous forests. The bordering seas contribute to diverse and transitional weather patterns; however, the overall region is characterized by hot (27 °C) and moderately rainy summers, and cold (0–4 °C) snowy or rainy winters, with an average annual precipitation of around 700 mm. In the entire study region, the population is 1,922,695 (around 70–75% in urban areas), the surface area is 21,108 km^2^, and population density is 91.1 per km^2^.

### Field study and sample collection

Field studies were carried out from 2016–2018, during the temperate and warm months of the spring and summer. Thirty-two sampling sites belonging to fourteen districts of Istanbul, Tekirdag, Kirklareli and Edirne provinces were visited (Fig 2b, Table 1). The tortoises were captured by hand during grazing within their natural environment and were identified for sex and species using taxonomic keys [20], and all parasitizing ticks from each animal were collected and placed in separate labeled vials. Blood samples were collected in heparinized tubes *via* the subcarapacial venous sinuses of tortoises ≥ 10 cm of straight carpal length (SCL; the straight-line measurement from the outermost projection of the nuchal plate to the posterior end of supracaudalia) following previously published protocols [29]. Questing, unfed adult ticks were collected by direct inspection/hand-picking and immature stages were collected by dragging at each sampling site. All samples were transported under cool conditions to the laboratory on the day of sampling, and during this period no specific medium was used. The tick stage, sex and species were identified according to taxonomic keys [14], before storage at -80 °C.

**Table 1:** Location data of study sites in East Thrace, Turkey

Site No.	Locality	District, Province	Coordinates (altitude)	Altitude (m)
1	Cemetery of the district	Catalca, Istanbul	41° 08′ N, 28° 27′ E	110
2	Pond of Sinekli	Silivri, Istanbul	41° 14′ N, 28° 11′ E	200
3	Cemetery of K. Kilicli	Silivri, Istanbul	41° 07′ N, 28° 12′ E	82
4	Cemetery of Gumusyaka	Silivri, Istanbul	41° 02′ N, 28° 02′ E	42
5	Cemetery of Sultankoy	Marmaraereglisi, Tekirdag	41° 01′ N, 27° 59′ E	17
6	Cemetery of Yeniciftlik	Corlu, Tekirdag	41° 00′ N, 27° 51′ E	71
7	Cemetery of the district	Suleymanpasa, Tekirdag	41° 58′ N, 27° 31′ E	38
8	Cemetery of Kumbag	Suleymanpasa, Tekirdag	40° 51′ N, 27° 27′ E	35
9	Cemetery of the district	Muratli, Tekirdag	41° 10′ N, 27° 29′ E	87
10	Cemetery of Misinli	Corlu, Tekirdag	41° 18′ N, 27° 36′ E	82
11	Field of Kastro	Saray, Tekirdag	41° 35′ N, 28° 08′ E	40
12	Cemetery of B. Karistiran	Luleburgaz, Kirklareli	41° 17′ N, 27° 32′ E	75
13	Field of Evrenli	Vize, Kirklareli	41° 31′ N, 27° 51′ E	170
14	Cemetery of the district	Vize, Kirklareli	41° 34′ N, 27° 46′ E	224
15	Field of the district	Vize, Kirklareli	41° 34′ N, 27° 47′ E	230
16	Field of Komurkoy	Vize, Kirklareli	41° 38′ N, 27° 52′ E	195
17	Cemetery of Kiyikoy	Vize, Kirklareli	41° 37′ N, 28° 05′ E	47
18	Field of Armagan	Central district, Kirklareli	41° 52′ N, 27° 25′ E	441
19	Field of Duzorman	Central district, Kirklareli	41° 50′ N, 27° 22′ E	401
20	Field of Korukoy	Central district, Kirklareli	41° 51′ N, 27° 19′ E	482
21	Field of Kuzulu	Central district, Kirklareli	41° 52′ N, 27° 16′ E	382
22	Field of Devletliaagac	Kofcaz, Kirklareli	41° 58′ N, 26° 59′ E	409
23	Field of Cayirli	Central district, Kirklareli	41° 52′ N, 27° 02′ E	245
24	Field of Karahamza	Central district, Kirklareli	41° 50′ N, 27° 02′ E	376
25	Field of Yoguntas	Central district, Kirklareli	41° 50′ N, 27° 03′ E	453
26	Cemetery of Yoguntas	Central district, Kirklareli	41° 49′ N, 27° 04′ E	333
27	Field of Kayali	Central district, Kirklareli	41° 48′ N, 27° 05′ E	299
28	Cemetery of Arpac	Havsa, Edirne	41° 41′ N, 26° 53′ E	118
29	Cemetery of Karaagac	Central district, Edirne	41° 38′ N, 26° 32′ E	36
30	Cemetery of Osmanli	Havsa, Edirne	41° 35′ N, 26° 50′ E	83
31	Cemetery of the district	Havsa, Edirne	41° 32′ N, 26° 49′ E	91
32	Cemetery of Kircasalih	Uzunkopru, Edirne	41° 23′ N, 26° 48′ E	113

### Viral RNA isolation and nested PCR assay

The tick samples collected from tortoises were pooled according to the site, and the stage, engorgement status, and sex (in most samples) of the ticks. Questing, unfed adult ticks collected from the ground were examined individually and immature questing stages were pooled according to the stages (larvae and nymphs). The tick samples were homogenized in liquid nitrogen and then total RNA was extracted using the E.Z.N.A®Viral RNA Kit (Omega Bio-Tech, Georgia, USA) following the manufacturer’s instructions. Extracted RNA samples were stored at -80 °C until use. The Qiagen®OneStep RT-PCR Kit (Qiagen, Hilden, Germany) was used in the first step of the nested PCR with the primers Eecf-F1 (5′-TTG TGT TCC AGA TGG CCA GC-3′), Eecf-R1 (5′-CTT AAG GCT GCC GTG TTT GC-3′), Eecf-F2 (5′-GAA GCA ACC AAR TTC TGT GC-3′), Eecf-R2 (5′-AAA CCT ATG TCC TTC CTC C-3′), to amplify a 211-bp fragment of the small (S) segment of CCHFV [30].

The RNA samples extracted from ticks and blood were processed within a Biosafety level 4 laboratory at the Galveston National Laboratory (Galveston, Texas, USA), using a CP02 cryoPREP automated dry pulverizer (Covaris, Woburn, MA, USA) on frozen and weighed tick samples. Homogenates were resuspended in Hanks balanced salt solution (Corning, Manassas, VA, USA) supplemented with 2% fetal bovine serum and 1% penicillin/streptomycin (Invitrogen, Carlsbad, CA, USA). Resuspended homogenates were added in a 1:5 ratio of Trizol reagent (Invitrogen), and RNAs were extracted using Direct-zol RNA miniprep kit (Zymo Research, Tustin, CA, USA), quantified, and first-strand cDNA synthesis was accomplished following SuperScript III Reverse Transcriptase kit instructions (Invitrogen). Amplification of first-strand cDNAs was accomplished using Phusion High-Fidelity DNA polymerase kits (New England Biolabs, Ipswich, MA, USA) using previously listed primer sets, along with laboratory created pan-CCHFV primer sets (available upon request by contacting D. Bente). Nested PCR of amplified products was run for optimization and/or verification purposes using DreamTaq PCR master mix kit (Thermo Fisher Scientific, Waltham, MA, USA) with clade-specific CCHFV primer sets within the pan-primer set amplified regions. Amplified fragments from both PCR and the nested PCR reactions were extracted using Zymoclean gel DNA recovery kit (Zymo Research, Irvine, CA, USA), and Sanger sequencing was performed *via* the UTMB Molecular Genomics Core (Galveston, Texas, USA). Sequenced products were compared to an in-house curated library of GenBank available CCHFV sequences using Geneious R11 (Auckland, New Zealand).

### Phylogenetic analysis of CCHF virus

PCR and nested PCR products were purified using polyethylene glycol (PEG) and the concentrations were determined photodensitometrically by comparison with a DNA standard using Bio1D [31]. For the phylogenetic analysis of purified DNA according to CCHFV S segment, the automatic sequence analysis method based on the Sanger method was used.

Purified DNA was bidirectionally sequenced and analyzed using GenomeLab DTCS—Quick Start DNA Sequencing Kit (GenomeLab DTCS, California, USA), using the Beckman Coulter GenomeLabGeXP Genetic Analysis System version 10.2 program. For the alignment of sequences, BioEdit version 7.2.5 was used [32]. The sequences were compared with the CCHFV sequences in the GenBank database. As outgroups, Dugbe virus (GenBank: FJ392604.1) and Hazara virus (GenBank: KP406725.1) were chosen. The number of tree generations was 1 million with partitions of frequency ≥ 0.10 in at least one run. The average standard deviation of split frequencies was 0.043382 and the maximum standard deviation of split frequencies was 0.237352. The average potential scale reduction factor (PSRF) for parameter values (excluding NA and > 10.0) was 1.026 and the maximum PSRF for parameter values was 1.576. The evolutionary history was inferred by using the Maximum Likelihood method based on the Kimura 2-parameter model [33]. The tree with the highest log-likelihood (−1913.02) is shown. The percentage of trees in which the associated taxa clustered together is shown next to the branches. Initial trees for the heuristic search were obtained automatically by applying Neighbor-Join and BioNJ algorithms to a matrix of pairwise distances estimated using the Maximum Composite Likelihood (MCL) approach and then selecting the topology with a superior log-likelihood value. The analysis involved 50 nucleotide sequences. All positions containing gaps and missing data were excluded; the final dataset contained a total of 212 positions. Evolutionary analyses were conducted in MEGA X [34].

### Study sites maps

Maps were created using ArcGIS® software by Esri (https://www.esri.com). ArcMap™ version 10.7 was used to create an overview map, inset map, proportional symbol map and relative proportion maps using the data supplied in Additional file 1: Tables S1 and S2.

## Results

### Tick infestation characteristics in the tortoises

During the study period, 71 feral adult tortoises from 32 different sites were examined for tick infestation in rural and urban parts of East Thrace (Table 2). Of those 71 tortoises, 65 [91.55%; 5 *T. hermanni* (♀) and 60 *T. graeca* (14♂, 46♀)], were infested with ticks. All the ticks found on the tortoises were identified as *H. aegyptium*. The biggest tortoise infested with ticks was a *T. graeca* female (at site 6, in May 2017) with 24.05 cm SCL. No ticks were found on tortoises with an SCL smaller than 10 cm (juveniles) exception for one specimen of *T. graeca* (from site 7, in May 2018) with an SCL of 7.09 cm from which 3 nymphs were collected. In total, 753 ticks (448 males, 152 females, 93 nymphs and 60 larvae) were collected from the animals. The counts of infested animals by male, female, nymphal and larval ticks were 57, 46, 16 and 9, respectively, and the counts of male, female, nymphal and larval stages per infested tortoise varied between 1–24, 1–12, 1–17 and 1–15, respectively. The greatest tick count was observed on a female *T. graeca* (at site 7); from this individual, 24 males, 5 females and 3 nymphs were collected in total. The study was performed in spring, summer and autumn seasons, with all three life stages (larva, nymph and adult) of *H. aegyptium* found in all three seasons. Co-feeding occurring among the different life stages of the ticks was frequently encountered. Although the immature stages of this species were found in the rostral part and adult stages were found in the caudal part of tortoises, this was not canonical, especially for adults which could feed in the frontal part of the body together with immature ticks (Fig. 1a, b). On ten tortoises, larval, nymph and mature stages of *H. aegyptium* were found as they were feeding simultaneously on the same animals. On seven tortoises, larvae were found with other stages, while on two tortoises they fed alone. On fourteen tortoises, nymphs were found with other stages, and on two tortoises they were found alone.

**Table 2:** Overview of *H. aegyptium* tick stages and blood samples collected from infested tortoises

Site no.	Sample no.	No. of tortoises screened (species/sex)	Samples collected from the tortoises		
			No. of ticks^a^	No. of tick pools	No. of blood samples
1	1	2 (*T. graeca* /♀)	20M, 11F	2	–
2	2	1 (*T. graeca* /♀)	3M	1	1
	3	1 (*T. graeca* /♀)	1M, 1F, 1N, 10L	4	1
	4	1 (*T. graeca* /♀)	1M, 2F	2	1
	5	1 (*T. graeca* /♀)	4M, 11F	2	–
	6	1 (*T. graeca* /♂)	3M, 1F, 5N	2	–
	7	1 (*T. graeca* /♀)	2F, 2N	2	–
3	8	1 (*T. graeca* /♀)	2M	1	1
4	9	1 (*T. graeca* /♂)	4M, 3F	1	–
	10	1 (*T. graeca* /♂)	1M, 2F	1	–
5	11	1 (*T. graeca* /♀)	19M, 3F	2	1
	12	1 (*T. graeca* /♀)	1M, 5F	1	–
6	13	1 (*T. graeca* /♀)	11M	1	1
	14	1 (*T. graeca* /♀)	8M, 5F	2	1
	15	1 (*T. graeca* /♀)	24M, 1F	2	1
	16	1 (*T. graeca* /♀)	12M, 2F	2	1
	17	1 (*T. graeca* /♀)	10M, 3F	2	1
	18	1 (*T. graeca* /♀)	21M	1	1
7	19	1 (*T. graeca* /♂)	1M, 1F	2	1
	20	1 (*T. graeca* /♀)	12M, 3F	2	1
	21	1 (*T. graeca* /♀)	24M, 5F, 3N	3	1
	22	1 (*T. graeca* /♀)	10M, 6F	2	1
	23	1 (*T. graeca* /♀)	3M, 5N	2	–
	24	1 (*T. graeca* /♀)	1F, 13N, 2L	3	–
	25	1 (*T. graeca* /♀)	3M	1	–
	26	1 (*T. graeca* /♂)	3M, 2F	2	–
	27	1 (*T. graeca* /♀)	1M	1	–
	28	1 (*T. graeca* /♂)	3M	1	–
	29	13 (*T. graeca* /7♀,6♂)	78M, 21F, 36N	19	–
8	30	1 (*T. graeca*/♂)	5M	1	–
9	31	2 (*T. graeca* /♀)	33M, 22F	2	–
10	32	1 (*T. graeca* /♀)	9M, 2F	1	–
11	33	2 (*T. graeca* /♀)	3M, 3N	2	–
12	34	2 (*T. graeca* /♀)	34M, 7F	2	–
13	35	1 (*T. graeca* /♀)	8M, 3F	2	–
14	36	1 (*T. graeca* /♀)	11M, 6F, 5N, 1L	4	1
15	37	1 (*T. graeca*/♂)	2M	1	–
18	38	1 (*T. graeca* /♀)	7M, 2F	2	–
23	39	2 (*T. graeca* /♀)	8M, 4F	3	–
25	40	1 (*T. graeca* /♀)	5M, 3F, 1N, 7L	4	1
	41	1 (*T. graeca* /♀)	17M, 10F	2	1
	42	1 (*T. hermanni* /♀)	1F, 5N, 5L	3	1
26	43	1 (*T. hermanni* /♀)	5L	1	–
28	44	1 (*T. hermanni* /♀)	1F, 15L	2	1
	45	1 (*T. hermanni* /♀)	6N, 5L	2	–
29	46	1 (*T. graeca* /♀)	17M	1	1
31	47	1 (*T. graeca* /♀)	10L	1	–
32	48	1 (*T. hermanni* /♀)	8N	1	–
Total		65 (5 *T. hermanni*, 60 *T.graeca*)	753 (448M, 152F, 93N, 60L)	106	21
14	49	Questing ticks collected from the sites	6N, 125L	5	
16	50		1M, 50L	2	
17	51		1N, 6L	2	
18	52		12M, 5F	17	
19	53		3M	3	
20	54		5M, 3F	8	
21	55		1M	1	
22	56		1F	1	
23	57		2M, 3F	5	
24	58		1F	1	
25	59		2L	1	
27	60		1M	1	
28	61		4L	1	
Total			312 (25M, 13F, 8N, 266L)	52	

^a^Data broken down by sex and life stage

*Abbreviations*: M, male; F, female; N, nymph; L, larva

### CCHFV genome detection in tick and tortoise samples

During the study period, 21 blood samples from different tortoises (2 *T. hermanni* and 19 *T. graeca*), 106 tick pools (containing 448 males, 152 females, 93 nymphs and 60 larvae) collected from 65 tortoises (5 *T. hermanni* and 60 *T. graeca*), 38 adult unfed questing ticks (25 males and 13 females, screened individually) and 14 pools (containing 8 nymphs and 266 larvae) of immature unfed questing ticks collected from the ground, were screened by nested PCR in total. As a result of the screening of these 179 samples, 17 (9.5%) were detected as positive as follows: 2 of 21 blood samples (9.52%); 13 tick pools from 12 separate tortoises (containing 18 nymphs in 3 pools, and 52 males and 8 females in 10 pools) of 106 tick pools from tortoises (12.26%); and 2 of 38 adult questing ticks (5.26%). No positive result was determined in 14 pools of immature questing ticks. In site 7, where the positivity was highest, 38 tick pools (containing 138 males, 39 females, 57 nymphs and 2 larvae) and 4 blood samples taken from 23 tortoises were screened, and of those, 8 tick pools (21.05%) were positive, taken from 7 tortoises (30.44%), but no blood positivity was determined in this site. Four tick pools (8 males, 5 females, 1 female and 15 larvae) consisting of newly attached, unfed ticks collected from two tortoises which were determined as blood-positive were negative, possibly as a result of their recent attachment. One pool was positive for each of the pools taken from the three tortoises whose blood samples were determined negative for CCHFV (Fig. 2d, e; Table 3).

**Table 3:** Overview of samples positive for the CCHFV genome by RT-PCR

Site No.	Sample No.	Date	Origin of the positive samples	Screened samples/pools from the positive origins	Result
2	6	June 2016	*T. graeca* (♂)	5N	+
				3M, 1F	–
	7		*T. graeca* (♀)	2F	+
				2N	–
5	11	May 2017	*T. graeca* (♀)	Blood	–
				3F	–
				19M	+
6	14	May 2017	*T. graeca* (♀)	Blood	+
				8M	–
				5F	–
	18		*T. graeca* (♀)	Blood	–
				21M	+
7	20	May 2017	*T. graeca* (♀)	Blood	–
				3F	+
				12M	–
	23	July 2016	*T. graeca* (♀)	3M	+
				5N	+
	24		*T. graeca* (♀)	1F	+
				13N	–
				2L	–
	25	April 2018	*T. graeca* (♀)	3M	+
	26		*T. graeca* (♂)	3M, 2F	+
	27		*T. graeca* (♀)	1M	+
	28		*T. graeca* (♂)	3M	+
28	44	July 2017	*T. hermanni* (♀)	Blood	+
				1F	–
				15L	–
32	48	July 2017	*T. hermanni* (♀)	8N	+
20	54	June 2016	Field (questing, unfed ticks)	1M	+
				1F	+
				4M^a^	–
				2F^a^	–

^a^Tick samples that were screened individually for the presence of the CCHFV genome

*Abbreviations*: M, male; F, female; N, nymph; L, larva; +, RT-PCR positive; –, RT-PCR negative

**Fig. 3:**
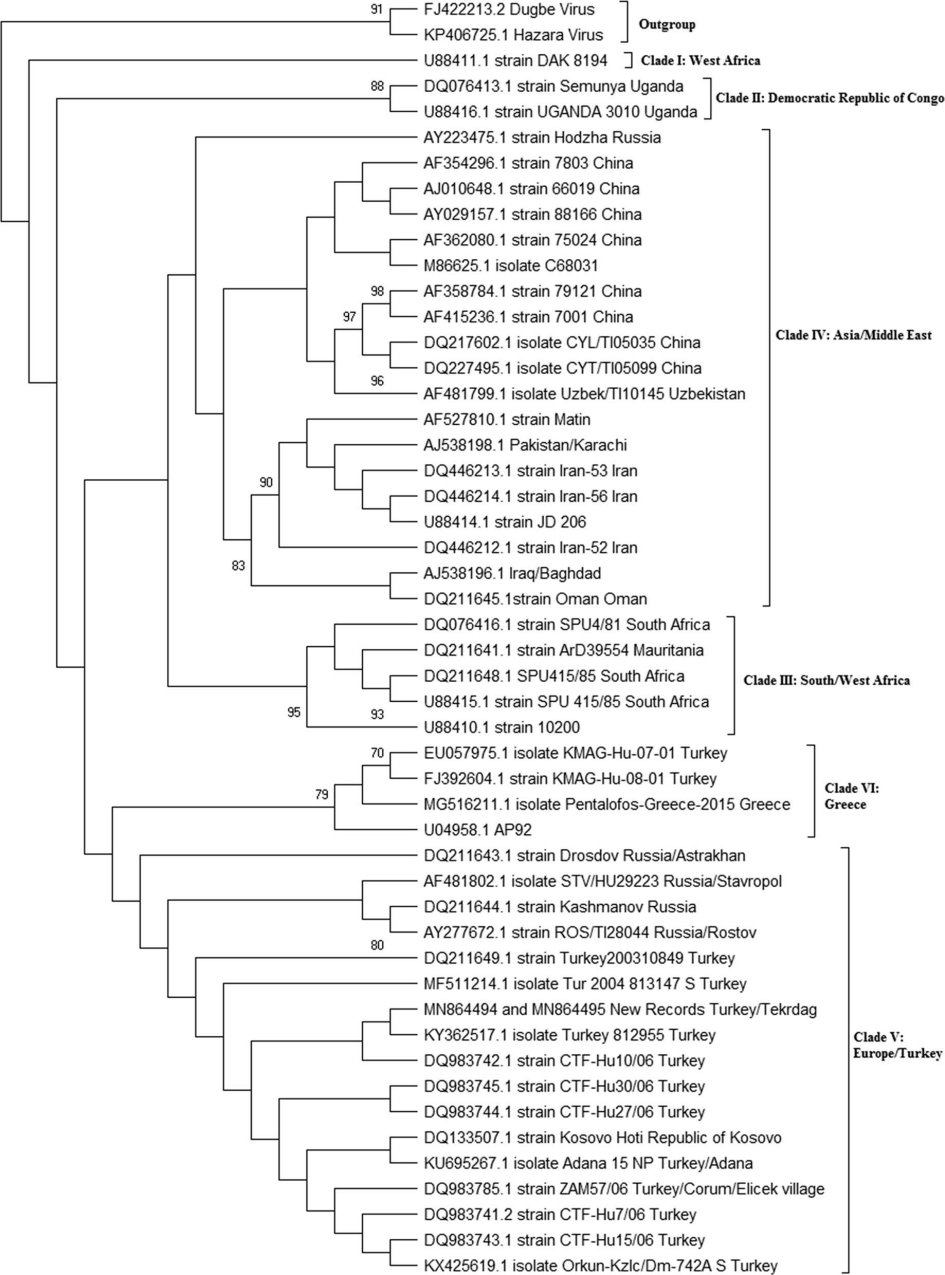
Phylogentic tree for CCHFV sequences based on a 211-bp fragment of the S-segment generated from tick and tortoise samples. CCHFV Clade V (Europe 1) was detected in tick pools collected from tortoises and in tortoise blood samples

In this study, we detected CCHFV (Clade V/Europe 1) in tick pools collected from tortoises (13/106; 12.26%), and in tortoise blood samples (2/21; 9.52%). Sequences are available under GenBank accessions numbers MN864494 and MN864495, respectively. This result indicates that this virus strain is possibly circulating at least among the tortoises and *H. aegyptium* in East Thrace. As a result of analyses of the 211-bp sequence of the virus S-segment, we detected a 2–5 bp difference between the present virus sequence and other sequences belonging to clade V virus on GenBank reported from *H. marginatum* from Turkey and Kosovo (Fig. 3).

## Discussion

Recent reports have demonstrated the presence of CCHFV genomic material in *H. aegyptium* [24–27]. This raises the question as to whether these ticks and their hosts play a role in maintaining CCHFV in nature. Unfortunately, the existing data is geographically and ecologically limited, and assessing the relevance of *H. aegyptium* and their hosts in perpetuating CCHFV in nature, and a potential spillover to humans, remains difficult. Here, we address this question for the East Thrace region in Turkey, where *H. aegyptium* is the most common human-biting tick species.

**Fig. 4:**
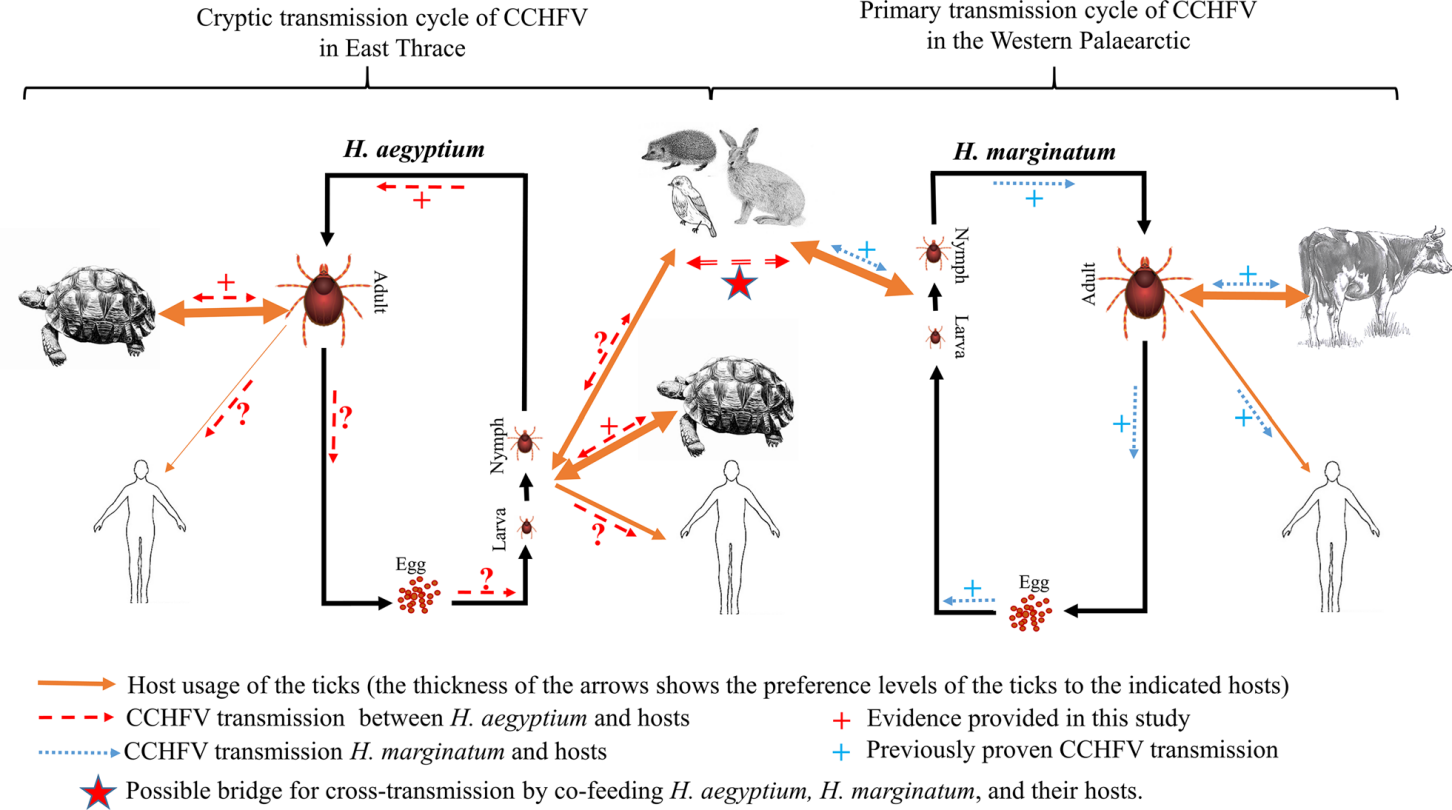
Proposed cryptic CCHFV transmission cycle in *H. aegyptium* and their hosts in East Thrace and the well-described primary CCHFV transmission cycle in *H. marginatum* and their hosts in the Palaearctic

In this study, we detected CCHFV in 12.26 % (13/106 pools) of the *H. aegyptium* tick pools collected from the tortoises, and in 5.26% (2/38 pools) unfed, adult questing field ticks. Importantly, the 13 positive tick pools were obtained from 12 separate tortoises (Table 3). The fact that these 13 positive tick pools were collected not from the same host but rather from many different hosts indicates that CCHFV prevalence is widespread and not that one tortoise is the super-transmitter for multiple tick pools. The percentage determined in the ticks collected from tortoises is within the range of other similar studies. In a study conducted in districts in Turkey and Syria on the east-north coastal area of the Mediterranean Sea, 245 adult *H. aegyptium* collected from 38 *T. graeca* were screened using RT-PCR; this study revealed a prevalence of 30.2%, and the sequenced virus was reported to be in clade III (Africa-3) [24]. In Algeria, 56 adult *H. aegyptium* collected from 12 *T. graeca* tortoises were tested for the virus, and 16 (28.6%) ticks were determined as positive for CCHFV, with 98–100% identity to the AP92 strain (Clade VI/Europe-2) [25]. The fact that the CCHFV genome can be found in unfed, questing ticks is evidence for a transstadial transmission of the virus (Fig. 4). Phylogenetic analysis of the partial S-segment indicated that all the sequences obtained from CCHFV-positive ticks as well as tortoises (see below) were 100% identical to each other and clustered in Clade V (Europe 1) (Fig. 3). The related analyses showed that sequences exhibited 97–99% identity with the other sequences reported from Turkey and Kosovo. This is the same clade as the currently circulating CCHFV strains in Turkey in *H. marginatum* ticks and their hosts. This might be an indicator that a tortoise-tick CCHFV transmission cycle is not an isolated cycle and actively connects with the CCHFV transmission cycle in *H. marginatum* (Fig. 4).

The CCHFV genome was detected in the blood of 2 out of 21 tortoises (9.5%). To our knowledge, this is the first report of CCHFV detection directly in tortoise blood. The viral sequence was similar to those discovered in *H. aegyptium* ticks, supporting the idea that ticks and hosts acquire CCHFV from each other. No virus isolation was attempted to compare the virulence of the CCHFV isolate from *H. aegyptium* to others. Considering that viremia in all vertebrate hosts experimentally tested is typically low-level and short-lived, there is a high probability that tortoises were exposed to CCHFV, yet, we missed the viremic window in the animals with our sampling. Unfortunately, no CCHFV antibody ELISA was performed on the blood samples to determine the percentage of seroconversion, although seropositivity does not always show productive replication of the virus or enzootic capacity of endemic hosts [35]. As previous serological surveillance demonstrates, reptiles are exposed to CCHFV by ticks feeding on them. In a study conducted on *T. horsfieldii* (Gray, 1844) in Tajikistan only one positive (1.7%) was determined from the 60 tortoises screened [36]. It is noteworthy to mention that all ticks collected in the present study from viremic tortoises were negative for CCHFV. This could be explained by the fact that the ticks were just recently attached and the long feeding period of *H. aegyptium* ticks. Alternatively, it is conceivable that the PCR-detectable viremia was on the decline or ultimately not high enough to infect the ticks collected from the animal. The concept that reptiles can be susceptible hosts and even long-term reservoirs for arboviruses is not new. For example, studies have demonstrated that alphaviruses and flaviviruses can be found in reptiles, and a bunyavirus has been isolated from the blood of a Texas soft-shelled turtle [37]. It is unclear how long the viremia persists and how can virus transmission between co-feeding ticks occur. Just as some species of other reptiles and mammals, species of the Testudinidae go through a period of hibernation. It remains unknown what happens to the virus during this phase of dormancy, and whether ticks feeding on an animal can become infected after the animal emerges from hibernation, therefore, serving as a virus reservoir. Studies of bats have demonstrated that viral infections can persist throughout hibernation for an extended period of up to 3 months [38]. Colorado tick fever virus has been shown to persist in golden-mantel ground squirrels (*Citellus lateralis*) throughout hibernation [39]. Thomas et al. [40] showed that the Western Equine Encephalomyelitis virus can overwinter in experimentally infected garter snakes and can be transmitted to mosquitoes, although viremia levels are expected to be low. Nevertheless, mathematical modeling efforts have revealed that arboviruses that adopt a low viremia and long persistence strategy have higher prevalence rates among both host and vector populations [41].

Although adult stages of *H. aegyptium* exhibit a high host preference and are almost exclusively found on testudinids, the immature stages of this species can also found on other hosts such as Lagomorpha, Erinaceinae and Aves (Fig. 4) [8, 42] which are also preferred by the immature stages of *H. marginatum*, a primary vector of CCHFV in the Western Palearctic [6, 10, 19] where both tick species can co-feed. Consequently, it is conceivable that the immature stages of *H. aegyptium* serve as bridge vectors between the well-described, primary CCHFV transmission cycle in *H. marginatum* and their hosts, and a cryptic CCHFV transmission cycle in *H. aegyptium* and the species of the Testudinidae. Recently, the importance of cryptic cycles for vector-borne diseases has been further understood and the number of relevant studies has increased. However, most of the studies are focused on mosquito-borne diseases [43–46], and as for cryptic cycles of tick-borne agents, only *Borrelia burgdorferi* has been studied in detail [47–50]. Cryptic cycles have been particularly emphasized in their contributions to enzootic pathogen maintenance in nature as its primary endemic importance particularly when cryptic and primary transmission co-exist [46, 49, 50]. In a comprehensive study recently performed in Tunisia, no CCHFV was detected in adult *H. aegyptium* specimens collected from *T. graeca*, and it was claimed that this tick species is unlikely to play a significant role in the epidemiology of CCHF [51]. This statement is not considering that there might be no bridging points in a cryptic and primary transmission cycle that allows spillover from one cycle into the other.

Our results raise the following questions about the cryptic cycle identified in this study: (i) What does the CCHFV replication in the tortoise host look like, and how effective is the transstadial and transovarial transmission of CCHFV in *H. aegyptium* ticks? (ii) How effective is the CCHFV co-feeding transmission of *H. aegyptium* and *H. marginatum* ticks on shared bridging hosts such as hedgehogs, hares and ground-dwelling birds? (iii) What is the risk of CCHFV transmission to humans through the bite of *H. aegyptium* immature life stages, and is the virulence attenuated in humans due to the adaptation in the *H. aegyptium*-tortoise cycle? (iv) To what degree can a potential cryptic CCHFV transmission cycle augment the primary cycle or even serve as a long-term reservoir due to the hibernation of tortoises? and (v) Could the cryptic tortoise cycle of CCHFV be of additional importance to the ecological, endemic, as well as the clinical characteristics of CCHF, apart from its possible ability to maintain the virus in its area?

Ongoing experimental studies will evaluate the CCHFV transmission dynamics between *H. aegyptium* and *Testudo* tortoises and address the questions above.

## Conclusions

In this study we show a high infestation rate of tortoises with *H. aegyptium* ticks in the East Thrace region of Turkey. We also demonstrate a high prevalence of the CCHFV genome in questing and feeding *H. aegyptium* ticks. To the best of our knowledge, this is the first report demonstrating the CCHFV genome in the tortoise hosts. *Hyalomma aegyptium* ticks are the most common human-biting ticks in the East Thrace region; therefore, this study raises the question of an additional CCHFV transmission route to humans. Our study may also indicate that CCHFV circulates in a cryptic transmission cycle in *H. aegyptium* and tortoise hosts that feed into the primary transmission cycle of *H. marginatum* ticks and their hosts and could play a role in the natural dynamic of the virus.

## Supplementary information

**Additional file 1: Table S1.** Overview of all the samples analyzed in the study for ARC GIS. **Table S2.** Overview of all the tortoise blood samples used in this study.

## Data Availability

All data generated or analysed during this study are included in this published article and its additional file.

## Abbreviations

CCHF: Crimean-Congo hemorrhagic fever
CCHFV: Crimean-Congo hemorrhagic fever virus
